# Influence of Environmental Factors on the Genetic and Chemical Diversity of *Brickellia veronicifolia* Populations Growing in Fragmented Shrublands from Mexico

**DOI:** 10.3390/plants10020325

**Published:** 2021-02-08

**Authors:** Yesenia Pacheco-Hernández, Nemesio Villa-Ruano, Edmundo Lozoya-Gloria, César Augusto Barrales-Cortés, Fabiola Eloisa Jiménez-Montejo, María del Carmen Cruz-López

**Affiliations:** 1 Centro de Investigación en Biotecnología Aplicada, Instituto Politécnico Nacional, Tlaxcala 90700, Mexico; yesenia.pachecoh@gmail.com (Y.P.-H.); fejimenezm@ipn.mx (F.E.J.-M.); 2 CONACyT-Centro Universitario de Vinculación y Transferencia de Tecnología, BUAP, Puebla 72570, Mexico; nemesio.villa@conacyt.mx; 3 Centro de Investigación y de Estudios Avanzados del IPN, Unidad Irapuato, Irapuato 36824, Mexico; 4 Universidad Iberoamericana Puebla, Puebla 72820, Mexico; cesar.barrales@iberopuebla.mx

**Keywords:** *Brickellia veronicifolia*, iPBS markers, essential oils, environmental factors

## Abstract

*Brickellia veronicifolia* is a native Asteraceae from Mexico that persists in fragmented habitats. This investigation reports the genetic and chemical diversity of *B. veronicifolia*. The diversity analysis based on iPBS markers showed an averaged Shannon index (*S*) of 0.3493, a Nei genetic diversity (h) of 0.2256, and a percentage of polymorphic loci average (*P*) of 80.7867%. The population structure obtained by AMOVA revealed that the highest variation found within populations was 94.58%. GC-MS profiling of six populations indicated that major volatiles were β–caryophyllene (11.63%), spathulenol (12.85%), caryophyllene oxide (13.98%), α–cadinol (7.04%), cubedol (6.72%) and tau-muurolol (4.81%). Mantel tests suggested a statistically significant relationship between minor volatiles and geographical distance (*r* = 0.6163; *p* = 0.0470; *p* ˂ 0.05). Likewise, major volatiles showed a significant correlation with the soil pH (*r* = 0.6862; *p* = 0.0230) and maximum temperature (*r* = 0.4999; *p* = 0.0280). Our study suggests that the variation and genetic divergence of *B. veronicifolia* has no relationship with climatic parameters, whereas the volatiles are probably influenced by environmental factors and not by the genotype per se. Based on the characteristics of *B. veronicifolia*, this plant could be considered as a candidate for restoring fragmented shrublands in Mexico.

## 1. Introduction

The environmental disturbance caused by anthropogenic activity is a critical factor that confines plant populations to fragmented habitats. As a consequence, genetic diversity is modified causing changes in the viability and adaptation of populations [1]. It is known that any ecological alteration produced by disturbance may generate unpredictable changes in the diversity and evolutionary processes of populations [2]. Environmental factors such as soil nutrients, temperature, water availability and light intensity influence the genetic and chemical diversity of plant populations. These environmental conditions can exert strong selection pressures, they could even determine the evolutionary course of plant populations [3,4]. This natural phenomenon causes that several plant populations constituted by a single species show different patterns of genetic and chemical variation in diverse geographical regions [5].

The maintenance of the genetic diversity of plant species with potential use for habitat restoration can improve the fitness of individuals and self-sustaining of populations [6]. Genetic constitution is essential in ecological restoration for understanding the adaptation of species to disturbed ecosystems [7,8]. Plants adapt to new environmental conditions mainly because of their genetic variability that may be related to specific chemical phenotypes [9,10]. 

**Figure 1:**
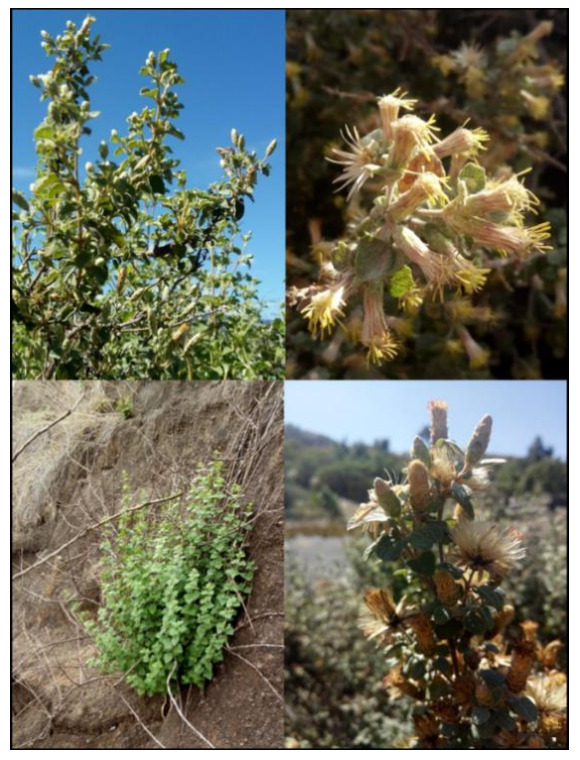
*Brickellia veronicifolia* in wild conditions.

*Brickellia veronicifolia* (Kunth) Gray (Figure 1), is an Asteraceae commonly known as “Peiston” or “estrellita dorada” in some provinces of Mexico. This medicinal plant grows in perturbed habitats and eroded soils of pine-oak forest, xerophytic shrubland and grassland [11,12]. *B. veronicifolia* is a perennial shrub that persists in habitats fragmented or perturbed by urbanization, contamination, overgrazing and agriculture (Figure S1). The plant exhibits interesting ecological characteristics such as drought and high temperature tolerance. The plant is able to grow under a wide altitudinal range (1500–3000 masl) [12]. Due to its ecological characteristics, this species is of interest for soil restoration in Mexico. Other species with similar characteristics such as *Buddleja cordata* y *Dodonaea* have been successfully used to recover degraded zones [13]. More than 50% of Mexican territory is comprised by xerophytic shrublands which unfortunately present a marked human footprint [14,15]. Due to this fact, plants with potential use in recovering perturbed habitats are highly required.

*B. veronicifolia* is an aromatic plant with interesting pharmacological properties which have been elucidated by several scientific investigations [11,16,17,18,19,20]. Previous phytochemical studies revealed methoxy-flavonols and labdane-type diterpenes as some bioactive compounds found in this plant [21,22,23,24]. 

However, there are no reports describing the genetic and chemical diversity of *B. veronicifolia* among wild populations grown in the Mexican territory. In this context, genotyping-based approaches are useful to know the genetic variability of plant species which is fundamental to understanding their population dynamics. Recently, iPBS (inter-Primer Binding Site) retrotransposons markers have been developed as an alternative method to explore genetic diversity and genetic relationships in “orphan crops” [25]. As a dominant marker system, iPBS fingerprinting is widely informative and reproducible and it does not require previous knowledge on the genome of a specific plant [26]. iPBS markers were designed under the premise that retrotransposons are abundant in coding and noncoding regions of eukaryotic genomes. Genotyping based on iPBS markers, has been successfully used to determine genetic variability in *Myrica rubra* [27], *Camellia sinensis* [28] and grapes (*Vitis vinifera* L.) [29], among other plant species. Considering the latter reasons, this marker was used in the present work to estimate genetic variability in *B. veronicifolia*.

On the other hand, chemical profiling is crucial for revealing particular differences in the biosynthesis of specific metabolites, which may be associated with specific geographical areas or genotypes [10]. The identification of factors involved in the genetic and chemical variation of native plant populations is useful for understanding interactions among biological and physical elements of the natural environment. This information could be critical to determine the potential utility of a particular plant for restoration aims. The correct interpretation of these data will significantly contribute to the restoration of ecosystems in the short term. It is known that fragmented and/or disturbed plant populations can provide valuable information on the effects they experienced at genetic or chemical level in order to understand their evolutionary response [30]. These data could be usable to predict the effects of genetic drift on some evolutionary forces such as gene flow (migration), changes in selective pressures and even to know if the populations are affected by inbreeding [31,32]. Most importantly, habitat restriction may affect the ecological interactions between plants and pollinators through changes in volatile biosynthesis [33]. Specifically, habitat fragmentattion will affect directly or indirectly both the genetic and chemical diversity of plant populations. Therefore, the aim of this study was to investigate the genetic and volatile variations of *B. veronicifolia* and their relationship with environmental factors associated with different fragmented sites within Mexico.

## 2. Results

### 2.1. Genetic Variability of B. veronicifolia Grown in Mexico

**Figure 2:**
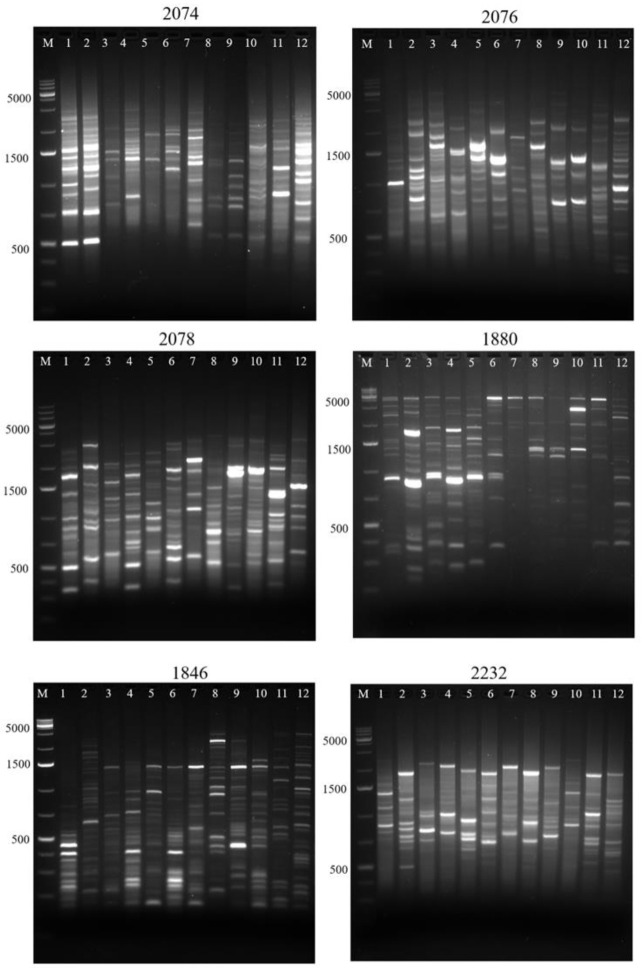
Inter-primer binding site (iPBS) based fingerprinting of *B. veronicifolia* (iPBS 2074, 2076, 2078, 1880, 1846 and 2232) (M = Molecular weight; 1–12 samples of Guanajuato population).

Genotyping results using iPBS markers showed a high genetic diversity associated to 360 different loci. This evidence revealed a diverse fingerprinting within the individuals from the six populations studied. The number of bands obtained were from 12 to 31 depending on the tested iPBS primer and the molecular weight oscillated between 250 and 5000 bp. The fingerprinting of Guanajuato samples using the 2074, 2076, 2078, 1880, 1846, and 2232 primers is shown in Figure 2. The genetic diversity estimators for each studied population are summarized in Table 1. The Guanajuato population showed the highest number of effective alleles (1.3818) and the highest Nei genetic diversity (*h*) value (0.2313). The highest value of observed alleles (1.8083) was found in the Hidalgo population whereas the Querétaro population exhibited the lowest *h* value (0.2233). The highest percentage of polymorphic loci (*P*%) was 80.50 for Oaxaca but the San Luis Potosí and Querétaro populations showed the lowest *P*% (79.44% and 79.72%, respectively). Shannon’s information index ranged from 0.3449 to 0.3568 with 0.3493 on average. The *FST* value for the total loci was 0.0335 (*p* ˂ 0.05) according to AMOVA. 

**Figure 3:**
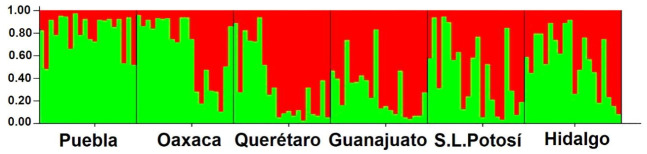
Population structure of 120 samples of *B. veronicifolia* for K = 2. Each vertical bar corresponds to a single sample and color proportion indicates a cluster produced by Structure 2.3.4 software.

**Figure 4:**
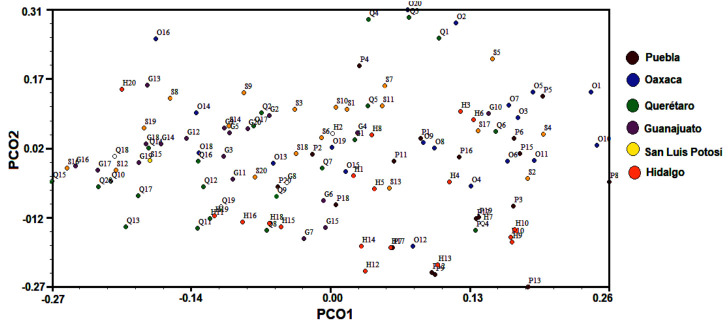
Two dimensional plot of the PCO-MC clustering analysis for the *B. veronicifolia* IPBS dataset.

**Figure 5:**
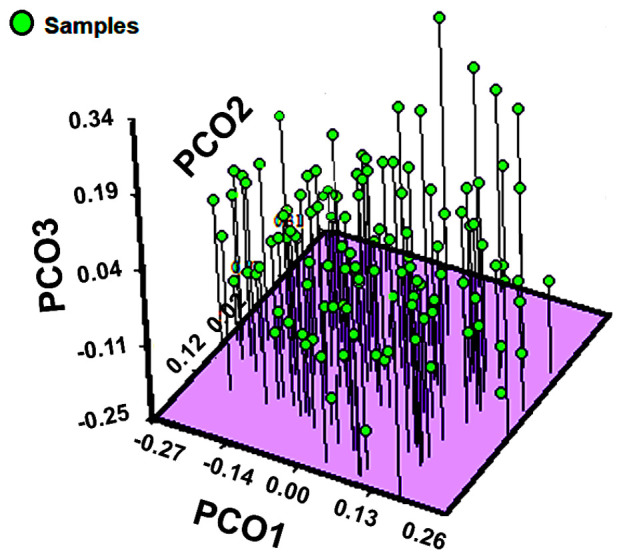
Three dimensional plot of the PCO-MC clustering analysis for the *B. veronicifolia* IPBS dataset.

The genetic structure analysis showed that the highest genetic diversity was found within populations (94.58%). The genetic variation among populations was 1.39% according to AMOVA approaches (Table 2). The Bayesian analysis of the genetic structure showed two subpopulations (K = 2) based on the maximum value of ΔK (Figure 3). Contrastingly, the results obtained from the principal coordinate analysis with modal clustering (PCO-MC) did not produce significant clusters. The corresponding 2D plot which was based on principal coordinates (PCO1 and PCO2) (Figure 4), showed that the studied individuals were not distributed in specific clusters. This result was clearly endorsed in the 3D projection (PCO1, PCO2 and PCO3) which revealed that all the samples belong to the same group (Figure 5). *FSC* (permuting haplotypes among populations within groups) and *FST* indexes applied to the obtained groups showed statistically significant (*p* ˂ 0.05) values of 0.04090 and 0.05423, respectively. 

The matrix of the genetic distances (*FST*) among the six populations of *B. veronicifolia* are shown in Table 3. The smallest genetic distance was between Hidalgo and San Luis Potosí populations (0.0153) whereas the greatest one was between Guanajuato and Puebla populations (0.0257). The averaged estimation of *Nm* (migrant number) for all populations was 12.565 and it was considered as an indirect estimation of the gene flow in *B. veronicifolia*. 

**Figure 6:**
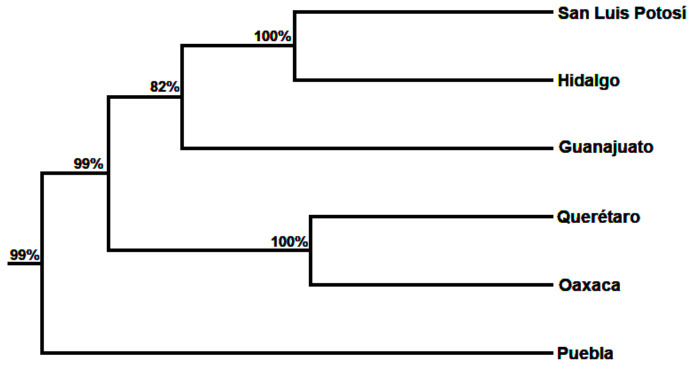
UPGMA dendrogram based on genetic distance (*FST*) using a bootstrapping of 1000 permutations.

The dendrogram obtained by the method unweighted pair group method based arithmetic means (UPGMA) showed two main population clusters which were genetically related (Figure 6). The first cluster was comprised of five populations (San Luis Potosí, Hidalgo, Oaxaca, Querétaro and Guanajuato) whereas the second one included the Puebla population. 

### 2.2. Volatile Profiling 

**Figure 7:**
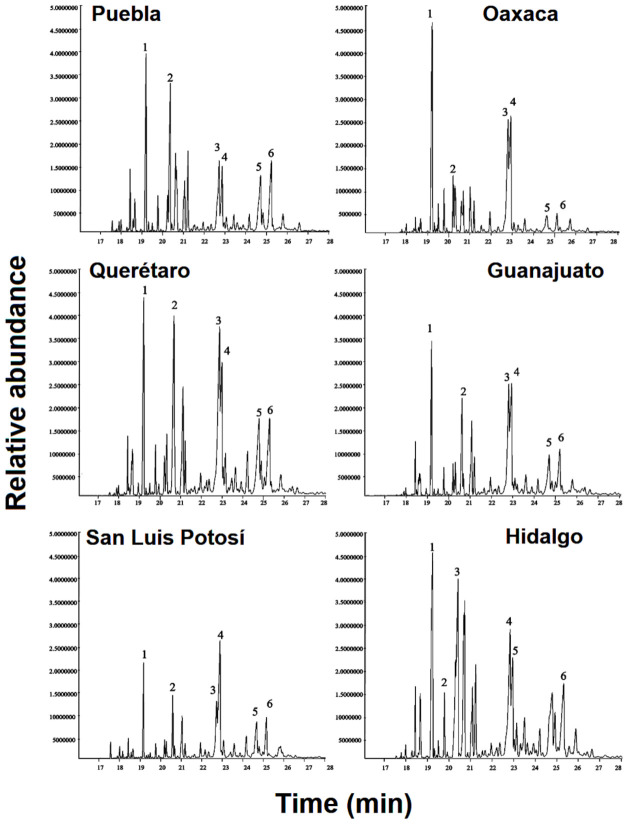
Total ion current of GC-MS showing the six major volatiles of the essential from *B. veronicifolia*. (1) β–caryophyllene, (2) cubedol, (3) spathulenol, (4) caryophyllene oxide, (5) tau-Muurolol and (6) α–cadinol respectively.

More than 100 volatiles (*n* = 108; mainly terpenoids) were identified in the leaf essential oils of *B. veronicifolia* and these metabolites were clustered into five groups. Nonoxygenated sesquiterpenoids were the most abundant (40.74%) followed by oxygenated sesquiterpenoids (33.33%), oxygenated monoterpenoids (18.52%), aliphatic hydrocarbons (6.48%), and nonoxygenated monoterpenoids (0.93%). Six compounds were the most abundant in all analyzed samples: β–caryophyllene (11.63%), spathulenol (12.85%), caryophyllene oxide (13.98%), α–cadinol (7.04%), cubedol (6.72%) and tau-muurolol (4.81%) (Figure 7). 

The San Luis Potosí population had 10 specific volatiles including (±)-lavandulol (0.08%), d-carvone (0.01%), (+)-longifolene (0.27%), β-patchoulene (0.30%), α-bergamotene (0.23%), γ-himachalene (0.37%), murolan-3,9(11)-diene-10-peroxy (0.46%), epiglobulol (0.88%), (-)-δ-cadinol (1.43%), and patchoulol (1.02%). The Oaxaca population had six volatiles including d-limonene (0.25%), β-chamigrene (0.76%), β-gurjunene (0.19%), (±)-cadinene (0.18%), di-epi-α-cedrene (0.05%), and isocaryophyllene (0.01%). The Hidalgo populations had cis-3-hexenyl valerate (0.06%), γ-gurjunene (0.01%), 3,7(11)-selinadiene (0.37%), γ-elemene (0.92%), and humulene-1,6-dien-3-ol (0.61%). The Querétaro population contained cis-2-p-menten-1-ol (0.02%), 5-caranol, trans-(+)-(0.01%), carotol (0.02%), and endo-borneol (0.03%). The Guanajuato population had three volatiles, these included ascaridole epoxide (0.01%), 7-epi-cis-sesquisabinene hydrate (0.04%) and guaiol (0.16%). The Puebla population contained only aristolene epoxide (0.14%) as a differential volatile (Table S1). 

**Figure 8:**
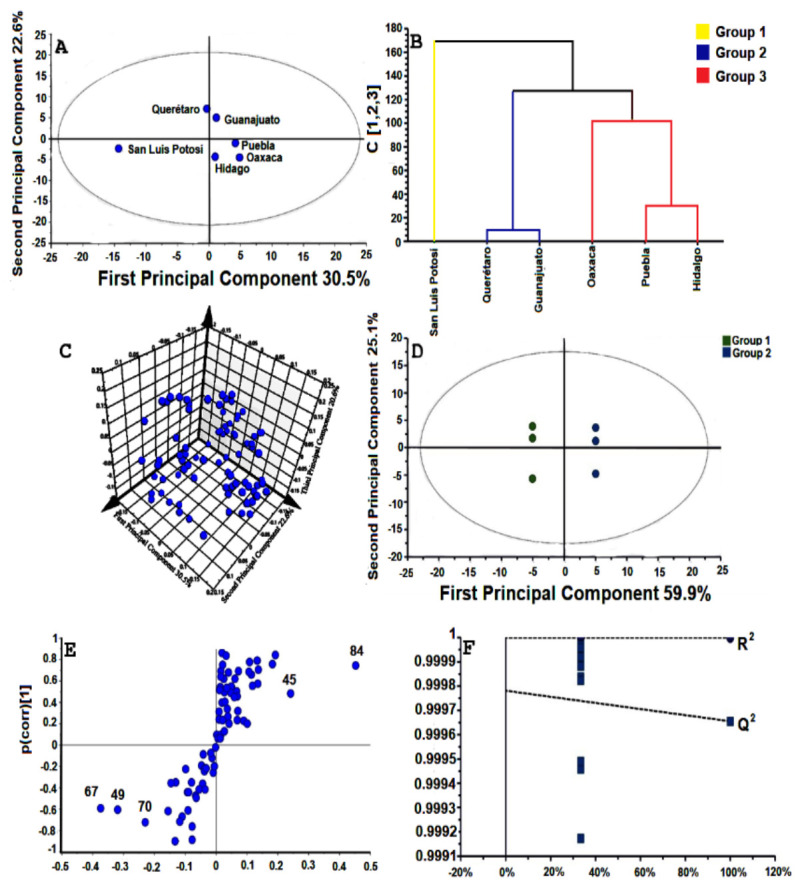
Chemometrics analysis of *Brickellia veronicifolia*. (**A**) Results of PCA from studied populations. (**B**) Dendrogram (HCA) based in PCA approaches. (**C**) Plot of 3D PCA. (**D**) OPLS-DA score plots generated from the volatiles of different populations of *B. veronicifolia.* Group 1, San Luis Potosí, Querétaro and Guanajuato; Group 2, Oaxaca, Puebla and Hidalgo. (**E**) S-plot discriminant analysis with potential chemical markers of *B. veronicifolia*. Caryophyllene oxide (84), β-elemene (45), β-caryophyllene (49), γ-cadinene (67), and (+)-δ-cadinene (70). (**F**) Statistical validation of the OPLS-DA model.

The principal component analysis (PCA) data produced a 2D projection [*PC1* = 30.5% and *PC2* = 22.6%] with a score plot showing high statistical values of *R*^2^*x* = 0.726 and *Q*^2^ = 0.256 (Figure 8A). The resulting plot revealed that San Luis Potosí was different from the other studied populations, and the hierarchical cluster analysis (HCA) indicated that it was chemically different from the others (Figure 8B). The 3D projections of the PCA model revealed a better separation between all analyzed volatiles [*PC1* = 30.5%, *PC2* = 22.6%, *PC3* = 20.6%] (Figure 8C). An orthogonal projection for latent structures discriminant analysis (OPLS-DA) model was applied to the total data set in order to identify a chemical correlation among the studied populations. Using one predictive component and three orthogonal components, this model was statistically significant (*p* < 0.05) with values of *R*^2^*x* = 0.850, *R*^2^*y* = 1.00, and *Q*^2^ = 0.999. The OPLS-DA model produced two groups which were effectively separated and chemically related, using projections into two dimensions (*PC1* = 59.9% and *PC2* = 25.1%). The first group was constituted by San Luis Potosí, Querétaro, and Guanajuato populations. The second one contained Oaxaca, Puebla, and Hidalgo populations (Figure 8D). The S-plot of this model was constructed to identify the metabolites differentiating the two groups. This approach revealed that caryophyllene oxide (84), β-elemene (45), β-caryophyllene (49), γ-cadinene (67), and (+)-δ-cadinene (70) significantly contributed to the separation of these groups (Figure 8E). The y-interception for *R*^2^ was 1.00 and for *Q*^2^ was 0.998. These parameters clearly validated the OPLS-DA model and suggested a high volatile variability in the wild populations of *B. veronicifolia* (Figure 8F). 

### 2.3. Environmental Data

The CONAGUA data showed that Guanajuato had 67.7 mm annual average precipitation and 19.6 °C temperature (Table 4). Both parameters were the highest for the populations studied. The physicochemical analysis of soil during the collection time revealed that Querétaro had the highest moisture (34.775%) and Puebla the lowest (8.769%). The soil pH was alkaline for Guanajuato (8.467) whereas acidic for San Luis Potosí (4.493). The phosphorus content was high for the soil from San Luis Potosí (0.150 mg/kg) and low for the soil of Querétaro (0.040 mg/kg). The carbon content was high in the samples from Hidalgo (3.225%) and low in the samples from Puebla (0.040%). Total nitrogen content was 50.933 mg/kg in soil samples from Oaxaca and 7.018 mg/kg in soil samples from San Luis Potosí. Finally, geographical data revealed that the altitude where plants were collected ranged from 1500 to 2480 masl (Table 5).

### 2.4. Correlations among Distance Measures

The Mantel Test performed with distance matrices of multiple variables revealed that the genetic distance did not have a significant statistical correlation with geographical distance among populations (*r* = 0.2216; *p* = 0.2980) nor with ecological distance (*r* = 0.4936; *p* = 0.0700). These results suggest that the geographical distance cannot explain the genetic divergence of wild *B. veronicifolia* populations. 

**Figure 9:**
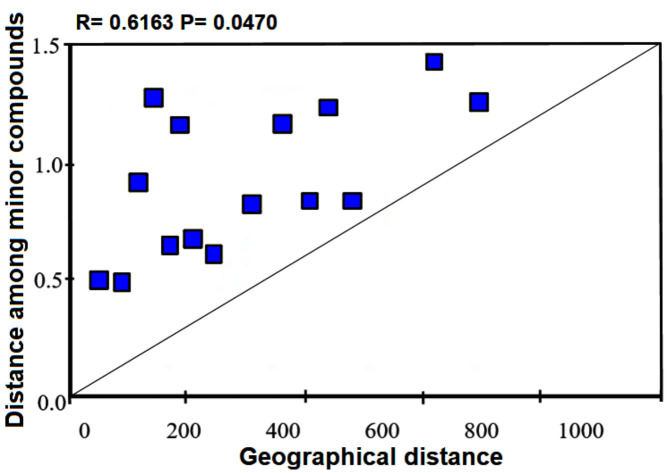
Correlation analysis using Mantel test between minor compounds and geographical distances (*p* ˂ 0.05).

Likewise, geographical distance did not show a significant correlation with environment factors (*r* = 0.2286; *p* = 0.2710) but it had a correlation with the distance matrix of latitude (*r* = 0.8051; *p* = 0.0160) and longitude (*r* = 0.6728; *p* = 0.0070). The genetically different populations showed a negative correlation with chemical distance indicating nonsignificant differences (*r* = −0.2116; *p* = 0.6750). On the other hand, analysis between minor compounds and geographical distance revealed a statistically significant correlation (*r* = 0.6163; *p* = 0.0470) (Figure 9). Similar results were found between the chemical profile and temperature (*r* = 0.3941 *p* = 0.0230). Specifically, minor volatiles showed a correlation with precipitation and temperature (*r* = 0.4305, *p* = 0.0460). Interestingly, the most abundant volatiles showed a correlation with pH (*r* = 0.6862; *p* = 0.0230) and maximum temperature (*r* = 0.4999; *p* = 0.0280). 

**Figure 10:**
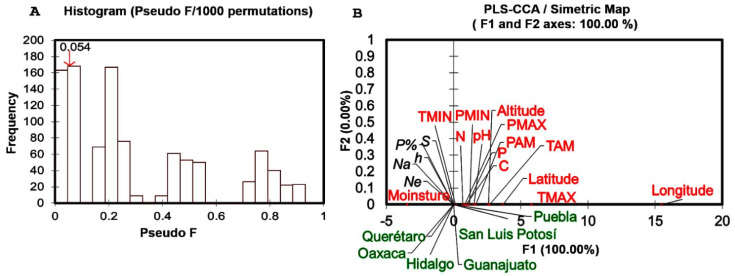
Partial least squares canonical correspondence analysis (PLS-CCA) of genetic diversity and environmental variables. (**A**) Histogram of permutation test. (**B**) Symmetric map of CCA among sites, genetic diversity and variables.

The results of partial least squares-canonical correspondence analysis (PLS-CCA) showed no significant values (*Q*^2^ = −0.328, *R*^2^*Y* = 0.000 and *R*^2^*X* = 0.725). Interestingly, the results of the permutation test (Pseudo *F* = 0.054; *p* = 0.824) revealed that genetic diversity was not linearly correlated with environmental variables (*p* > 0.05) (Figure 10A). These results were endorsed with inertia percentages (PLS-CCA) and eigenvalues. According to our results, 94.846% inertia was found in CCA-unconstrained and only 5.154% was associated to CCA-constrained, both presented eigenvalues of 0. The map of canonical correspondence analysis did not show any association among sites, genetic diversity and environmental variables (Figure 10B). 

## 3. Discussion

The estimators of genetic diversity for *B. veronicifolia* were generated by iPBS markers and were similar to those reported for populations of *Smallanthus sonchifolius* [34], motherwort (*Leonurus cardiaca* L.) [35] and *Cicer* sp. [36]. The high genetic diversity observed in different populations can be attributed to a high genetic flow. This asseveration is supported by the low *FST* value which is proportional to the low differentiation found in *B. veronicifolia* populations. The low differentiation among these populations suggests a closer connection among them which was additionally endorsed by *Nm* > 1. This value also suggests that genetic flow exceeds the effect of genetic drift [37].

The high genetic flow among populations could reflect an effective mechanism of seed dispersion. Despite no information having been previously reported for the dispersion *B. veronicifolia* seeds, our observations made under open field conditions and the morphological characteristics of the achenes (Figure S2), strongly suggest that anemochory should be associated to the seed dispersion of this plant as occurring in many species of the Asteraceae family [38].

The results of the genetic structure for *B. veronicifolia* estimated with STRUCTURE and PCO-MC were evidently divergent. The results obtained with STRUCTURE suggested two subpopulations (K = 2) within the population structure whereas those obtained by PCO-MC analysis revealed a single subpopulation. According to these approaches, it can be assumed that the studied populations of *B. veronicifolia* have no genetic structure. 

Although STRUCTURE software is widely used for this aim, it may present some limitations to differentiate populations with no genetic structure. Because of this, the results obtained from PCO-MC approach should be more than adequate to explain the genetic structure of the studied populations. The results of PCO-MC were consistent with the values obtained by AMOVA which revealed a higher genetic variation within the populations produced by the high rates of gene flow among the populations of *B. veronicifolia*. The grouping patterns of the UPGMA analysis differ from those obtained by PCO-MC. The UPGMA dendrogram, indicated that the Puebla population diverged from other populations. However, the Puebla population may be closely related to the Oaxaca population in accordance with the pattern showed in Figure 3.

The genetic diversity of the studied populations from *B. veronicifolia* did not show any signs of inbreeding. In the same context, the high genetic diversity of this plant supports an exogamic reproduction and suggests that the pollination process is highly efficient. Based on our open field observations, bees and butterflies could be involved in the pollination of *B. veronicifolia*. Previous reports on the pollinators of *Brickellia californica* may support this possibility [39]. Life history and selective pressures caused by climatic conditions may also influence the genetic variation of plant species [40,41,42]. Considering that iPBS markers are located in coding and non-coding regions of plant genomes, they can be modified by natural selection. Based on the latter premise, the high genetic variation found in the population from Guanajuato could probably be derived from different selection pressures associated with the human disturbance and also with changes of temperature and precipitation. Remarkably, this population showed the highest average temperature and precipitation compared with other populations of *B. veronicifolia*. This climatic variation may be related to the genetic diversification of the Guanajuato population. Despite the environmental factors did not show any statistical correlation with both the genetic diversity and genetic differentiation, we cannot completely assume that genetic variation was not modified by those factors.

A previous study suggests the possible effect of precipitation on the genetic diversity of *Rheum tanguticum* [43]. Other report describes the effect of precipitation on the genetic divergence of *Echinacea angustifolia* which is reflected in an increase of plant reproduction [42]. Based in our genetic data, the distribution of *B. veronicifolia* to a wide variety of environments can probably be linked to its genetic polymorphism. Although *B. veronicifolia* grows in fragmented environments and experience distinct types of ecological disturbances, these conditions apparently did not affect the intra-population genetic diversity of the plant. Similar effects have been observed in *Catopsis compacta*, which maintains a high genetic flow and a genetically diverse population even though it is strongly harvested with fragmentation and loss of its habitat [44]. Our study suggests that habitat fragmentation did not reduce the genetic diversity of *B. veronicifolia* and contrarily, it could be favoring seed dispersion and gene flow among populations. As a consequence, an increase in both genetic variability and persistence of *B. veronicifolia* in perturbed sites with particular climatic conditions could be favored.

### 3.1. Chemical Diversity and Population Differentiation 

The essential oils of *B. veronicifolia* revealed a high diversity of secondary metabolites (Table S1), however, it was not possible to identify specific chemotypes in the studied populations. Six major volatiles were constant in all populations representing around 61% of the total oil composition. Particularly, β–caryophyllene, spathulenol, and caryophyllene oxide constituted more than 40% of the essential oil. This volatile profile was quite different to that reported by Rivero-Cruz et al. 2006 [45]. Environmental factors such as collection time, geographical location, growth stage, genetics, biotic and abiotic conditions as well as methods of extractions, could be influencing differences in volatile profiling [5,46]. Intriguingly, the chemical profile reported in our work remained constant in all the studied populations of *B. veronicifolia* at the expense of environmental conditions and genetic constitution. The major sesquiterpenoids reported in our work have also been found in other Asteraceae plants such as *Centaurea tuberosa* [47] and *Eremanthus erythropappus* [48]. 

The PCA results showed that the San Luis Potosí population was chemically divergent from the other samples. It contained 70 different compounds (64% of the essential oil) and ten unique compounds. This result may be produced by physicochemical properties such as low pH (4.49), high P content (0.150 mg/kg), low C content (0.394%) and low N concentrations (7.018 mg/kg). The Mantel tests indicated that pH had a statically significant correlation. It is known that pH impacts on the availability of minerals such as nitrogen [49] and inorganic phosphorous [50]. It can also modify the charge and vapor pressure of volatiles [51]. Although the San Luis Potosí population did not experience high temperature rates, the correlation analysis indicated a direct link between maximum temperature and the synthesis of major volatiles. This population had the lowest annual precipitation (an average of 32.6 mm). It is known that drought stress stimulates the production of specific volatiles [46,52]. This fact can also modify the rate of herbivory [53,54] and the incidence of phytopathogens [55,56] in which volatiles have an evident role. Differences in precipitation rates and temperature confirmed an association with the volatile profiling of *B. veronicifolia*. 

The volatiles from *B. veronicifolia* were separated into two groups according to the content of monoterpenes and sesquiterpenes. The OPLS-DA model revealed that the geographical location was probably involved in the separation of both groups (Figure 8D,F). Chemometric analysis suggested that caryophyllene oxide and β-elemene are potential volatile markers due to their high magnitude and reliability. β-caryophyllene, γ-cadinene, and (+)-δ-cadinene were also critical for the separation of those groups (Figure 8E). Regarding their relative abundance, these compounds have been identified as chemical markers in other plant species such as *Salvia runcinata* [57], *Leonotis leonurus* [58], peels of citrus fruits [59], *Lantana radula* and *L. canescens* [60]. In the same context, 1, 8-cineole and β-caryophyllene were tagged as chemical markers and used to differentiate chemotypes for the essential oil of *Thymus serpyllum* [61]. 

Based on this background and in our statistical analysis, we found that β-caryophyllene, caryophyllene oxide, and spathulenol were constant in all populations studied. Thus, these compounds could be considered chemical markers for the volatile fraction of *B. veronicifolia*. In addition, the essential oil of this plant should be considered as a natural source of volatiles with anti-inflammatory, antiproliferative, analgesic, and antimicrobial properties [62,63]. 

### 3.2. Relationship among Genetic, Chemical, and Environmental Variables 

The correlation analysis between genetic and geographical distances showed no evidence of distance isolation among populations. These results suggest that the population are connected and not isolated. The genetic parameters including those of genetic flow, endorsed this finding. Simultaneously, it was observed that the genetic divergence of *B. veronicifolia* was not related to environmental differences. The results of the Mantel test exhibited that differences in latitude and longitude increases with geographical distance. It was also observed that an increase in geographical distance is directly proportional to the distance of minor compounds. On the other hand, the statistical relationship among major compounds, temperature, precipitation, and pH suggest that the synthesis of secondary metabolites is probably controlled by environmental factors. Although no statistical correlations between genetic distance and chemical distance were observed, our approaches cannot discard the involvement of the genetic constitution on the production of secondary metabolites. This asseveration is made on the fact that some loci amplified by iPBS are probably not directly linked to the expression of coding sequences associated with the biosynthesis of the studied volatiles. Although our results strongly suggest that environmental variables have a dominant role in volatile biosynthesis, the effect of biotic factors should be addressed in further studies.

### 3.3. Brickellia Veronicifolia and Its Implications in Restoration 

Our results suggest that *B. veronicifolia* showed a high genetic and chemical diversity at the intrapopulation level. These characteristics suggest that *B. veronicifolia* could be a species plenty adapted to human perturbation. In addition, in situ detection of younger specimens of *B. veronicifolia* suggests that perturbation may help to the fast reproduction of this species. Similarly to *B. veronicifolia*, other plants growing in disturbed and eroded soils could be potential candidates for recovering xerophytic shrublands affected by anthropogenic activity. 

The use of native species with high genetic and chemical diversity may increase the possibility of recovering perturbed ecosystems. The knowledge of genetic and chemical diversity of plants as shown in this work for *B. veronicifolia*, can be contemplated as complementary tools for land managers and conservation biologists in order to make the best decisions in restoration programs. For this task, knowledge on seed origin, genetic variation, and its relationship with environmental factors of specific sites, are highly required. Based on the genetic and chemical variability, our study suggests that the Guanajuato and San Luis Potosí populations are the best candidates for restoring xerophytic shrublands from Mexico and possibly other disturbed ecosystems where this plant is able to grow. 

## 4. Materials and Methods 

### 4.1. Plant Material 

**Figure 11:**
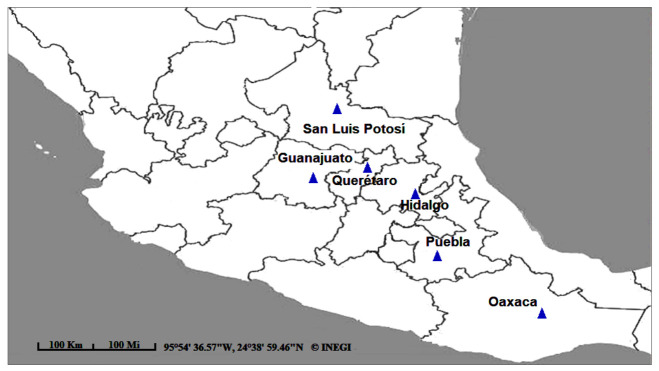
Geographical location of *Brickellia veronicifolia* populations grown in Mexico.

Young leaves of *B. veronicifolia* were collected from six different populations within Mexico (Puebla, Oaxaca, Querétaro, Guanajuato, San Luis Potosí and Hidalgo) (Figure 11). These populations were mainly associated to xeric shrublands which showed evidence of anthropogenic disturbance. Distance range among populations oscillated between 62 and 478 km. Based on these distances, the studied populations showed an evident isolation. Twenty plant samples were collected in each population studied and these were selected on the basis of their foliage and height (about 1 m). 

Samples were collected considering at least 3 m of separation each other. 

Voucher specimens were deposited at the Herbarium of the Faculty of Sciences, UNAM, Mexico (Table 5). The plant material was collected in summer 2017, transported at 4 °C to the laboratory and stored at −80 °C for further analysis.

### 4.2. DNA Extraction and iPBS Amplification

DNA was extracted from 100 mg of fresh leaves using the E.Z.N.A. ^®^Plant DNA DS Kit (Omega bio-tek, Norcross, GA, USA) according to the manufacturer’s instructions. The quality of the isolated DNA was confirmed by electrophoresis using a 1.0% agarose gel, final concentration was adjusted to 40 ng μL^−1^ for genetic analysis and stored at −20 °C for further PCR assays. Twelve iPBS (internal primer binding sequence) primers (Table S2) were used for genotyping [25,34]. PCR assays were performed in 12.5 µL reaction mixtures containing 1 unit of Platinum™ Taq DNA polymerase (ThermoFisher Scientific, Waltham, MA, USA), 2.0 mM MgCl_2_, 0.2 mM dNTPs (Invitrogen™, Carlsbad, CA, USA), 1 mM of 12–13-nt or 0.6 mM of 18-nt primers, and 40 ng genomic DNA. PCR were performed as follows: 1 denaturation cycle at 94 °C for 3 min followed by 35 cycles of 30 s denaturation at 94 °C, 45 s annealing at 49–60.5 °C, and 2 min 30 s extension at 72 °C. Final extension was for 5 min at 72 °C with a final step at 4 °C. PCR products were separated by electrophoresis at 100 V for 2.5 h in agarose gels 1.7% using 1X TAE buffer (0.04 M Tris-acetate, 0.001 M EDTA) using GeneRuler 1 Kb ladder (ThermoFisher Scientific, Waltham, MA, USA) to estimate fragment lengths. Gels were stained with GelRed (Biotium, Fremont, CA, USA) and recorded in a BIO-RAD Gel Doc™ XR equipped with an Image Lab™ Software.

### 4.3. Genetic Statistics Analysis

Well-defined DNA bands were categorized as present (1) or absent (0) using Image Lab™ software and carefully checked. Data were deposited in an Excel spreadsheet app as a binary matrix. The observed number of alleles (*Na*), number of effective alleles (*Ne*), Shannon’s information index (*S*), genetic diversity (*h*) [64], genetic differentiation between populations (*FST*), and polymorphism percentage (*P*%) were determined using POPGENE ver. 1.32 software [65]. All these calculations were made under the premise of that populations are in Hardy-Weinberg equilibrium. Genetic structure was examined using analysis of molecular variance (AMOVA) and 10,000 permutations with ARLEQUIN ver. 3.5.1 [66]. Three groups were well defined according to closer distances between pairs of populations (Group 1: Puebla and Oaxaca, Group 2: Hidalgo and Querétaro, Group 3: Guanajuato and San Luis Potosí). The genetic variation distribution was evaluated using the sum of squared size difference (*RST*-like) with 10,000 permutations. The genetic structure of the populations of *B. veronicifolia* was also evaluated by Bayesian clustering method using the STRUCTURE 2.3.4 software [67]. An Admixture model was used in order to include specimens in sub-populations (*K*). These approaches were done estimating their proportional pertinence by using a Correlated Allele Frequency model [68]. The range of *K*-values analyzed was from 1 to 12. The number of the algorithm MCMC was 100,000 and a burn-in length of 10,000 with 1000 iterations for each *K*-value was set. The optimal *K*-value was selected from the logarithm of maximum likelihood using the log probability of the data [LnP(D)] and delta *K* (Δ*K*) in accordance with the procedure described by Evanno et al. 2005 [69]. The data were processed by Structure Harvester [70]. 

Considering that STRUCTURE estimates the results using a constant number of populations, we additionally performed the PCO-MC approach described by Reeves and Richards [71] in order to contrast both results. This method is based on principal coordinate analysis (PCO) and clustering procedure to infer subpopulations in a dataset. 

PCO analysis was done with NTSYS 2.11 using Jaccard distances and DCENTER/EIGEN modules to compute principal coordinates along all axes. The cluster analysis was carried out using the MODECLUS procedure with SAS 9.1 (SAS Institute, Cary, NC, USA). Dendrogram were constructed with the means of the unweighted pair group method based arithmetic means (UPGMA) using MEGA X Software [72] and CONSENSE module in PHYLIP v3.69. The dendrogram was built using a distance matrix of *FST* values with a bootstrapping of 1000 permutations in AFLP-SURV ver. 1.0 [73].

### 4.4. Essential Oil Extraction and GC–MS Analysis 

Leaf essential oils were obtained by hydrodistillation (100 g leaves) using a Clevenger type apparatus for 3 h. The essential oils were collected in dichloromethane, dried over silica gel, and stored at 4 °C in amber glass vials until analysis. Essential oil yield was calculated on a moisture-free basis as 0.08–0.19% (*w*/*w*). GC-MS used an Agilent technologies 7820A instrument with a HP-5 capillary columns (30 m × 0.32 mm I.D. covered with a 0.25 µm of 5:95 phenyl-dimethylpolysiloxane plate). The mobile phase was helium at 1 mL min^−1^ flow rate and 180 °C injector temperature; the oven program was from 60 °C for 3 min, 75 °C for 5 min, 150 °C for 10 min, and up to 250 °C for 20 min. Software was set for 30–600 *m/z* range, and the mass spectra was recorded at 70 eV. The relative retention index (RRI) was calculated with a standard mix of C_8_–C_20_ *n*-alkanes (Sigma-Aldrich Co., St Louis, MO, USA) under identical conditions [74]. Data were compared with the NIST 2.0 database, with authentic d-limonene, β-linalool, β-caryophyllene, caryophyllene oxide, and (+) aromadendrene standards (Sigma-Aldrich Co., St Louis, MO, USA) and related literature [75,76]. The percentage of abundance was calculated from GC chromatograms. 

### 4.5. Chemometrics Analysis

The chemical relationship among the studied populations was analyzed by principal component analysis (PCA) to identify possible trends and outliers among these populations. A hierarchical cluster analysis (HCA) was constructed to observe data distribution and to explore the classification of samples. Orthogonal projection for latent structures discriminant analysis (OPLS-DA) was performed to identify differential metabolites among plant groups. For this analysis, a hoteling T^2^ region showing an ellipse in score plots of the model was considered using a 95% confidence interval. All variables were pareto scaled to minimize noise. Validation was done with the *R*^2^*x* and *Q*^2^ values using 999 permutations analysis and performed with SIMCA ver. 16.0 [77].

### 4.6. Environmental Data

In order to estimate environmental variability, altitude, latitude, longitude, averaged soil properties, temperature and precipitation rates were recorded for each studied population. These variables were selected because of their relationship with changes in genetic and chemical variation reported in previous works [32,42,78]. For Mantel test, the climatic annual data obtained in the years 2000–2017 (historical records) were averaged. These data were kindly provided by the National Water Commission of Mexico [79] (Figures S3 and S4). For calculations, only annual climatic variation was considered whereas monthly variation was not contemplated.

Analyses of soil properties were carried out in accordance with the Mexican norm NOM-021-SEMARNAT-2000. Briefly, soil samples were simultaneously obtained during the collection of plant material. Fifteen soil samples were manually extracted at 30 cm depth after defining a zigzag pattern within the sampling zone. Posteriorly, samples were mixed, homogenized and separated in fourth fractions until obtaining 1.5 kg soil [80]. Organic carbon (C), phosphorus (P), nitrogen (N), pH, and moisture of the upper soil layer were determined according to the same norm. 

### 4.7. Statistics 

Correlations between the genetic distance (*FST*), geographic distance (measured in kilometers), chemical distance, and climatic factors of populations were performed by Mantel tests with 999 permutations and a *p* ˂ 0.05 using TFPGA ver. 1.3 [81]. Data on soil properties as well as geographical, chemical, and genetic tests were standardized (z-score) before analysis to avoid side effects from different measurement units. For chemical data, major, minor, and all metabolites were used. Matrices containing geographical, chemical and environmental data were constructed from Euclidean distance using a dissimilitude measurement. This strategy was carried out in SPSS ver. 20.0 software in order to determine differences between pairs of populations [82]. Additionally, a PLS-CCA (Partial Least Squares-Canonical Correspondence Analysis) was performed to determine a possible correlation between genetic diversity and environmental variables. Considering the low number of observations PLS-model was used, this analysis was made with 1000 permutations at a significance level of *p* ˂ 0.05 using XLSTAT ver. 2020 software. 

## 5. Conclusions

This investigation reports for the first time the genetic and chemical diversity of wild populations of *B. veronicifolia* grown in Mexico. The Guanajuato and San Luis Potosí populations showed the highest genetic and chemical diversity, respectively. The genetic variation resulted from of a high gene flow among populations. This study revealed that the genetic divergence of *B. veronicifolia* does not depend on environmental variables. On the other hand, chemical diversity was probably influenced by environmental factors. Genotyping technique based on iPBS markers and chemometrics analyses were efficiently used in *B. veronicifolia*. Our findings suggest that *B. veronicifolia* has a significant potential for restoration and further strategies for their conservation and rational use should be addressed. However, the relationship of biotic factors (herbivory and microbial interactions) with genetic and chemical properties of *B. veronicifolia* populations should be further addressed.

## Figures and Tables

**Table 1:** Statistics for 120 individuals of *Brickellia veronicifolia* with 12 iPBS primers.

Populations	*Na*	*Ne*	*h*	*S*	*P*%
Puebla	1.8167	1.3721	0.2267	0.3519	81.67
Oaxaca	1.8250	1.3688	0.2239	0.3479	82.50
Querétaro	1.7972	1.3703	0.2233	0.3449	79.72
Guanajuato	1.8056	1.3818	0.2313	0.3568	80.56
San Luis Potosí	1.7944	1.3700	0.2241	0.3468	79.44
Hidalgo	1.8083	1.3682	0.2240	0.3474	80.83
Mean	1.8079	1.3719	0.2256	0.3493	80.78
St. Dev	0.0106	0.0046	0.0028	0.0040	1.0583

*Na*: observed number of alleles; *Ne*: number of effective alleles index; *h*: gene diversity; *S*: Shannon’s index; *P*%: polymorphism percentage.

**Table 2:** Analysis of molecular variance of groups based in the geographical location of *B. veronicifolia* populations.

Source of Variation	d.f	Sum of Squares	Variance Components	Percentage of Variation	Fixation Indices
Among groups	2	244.550	0.73646	1.39	*FCT*: 0.01390
Among populations within groups	3	278.450	2.13610	4.03	*FST*: 0.05423 *
Within populations	114	5710.800	50.09474	94.58	*FSC*: 0.04090 *
Total	119	6233.900	52.96729	100	

* *p* ≤ 0.05 *FST* (permuting haplotypes among populations among groups); *FSC* (permuting haplotypes among populations within groups); *FCT* (permuting populations among groups).

**Table 3:** Pairwise population matrix of *FST* based in Nei (1972) genetic distance for six wild populations of *Brickellia veronicifolia.*

	Puebla	Oaxaca	Querétaro	Guanajuato	San Luis Potosí	Hidalgo
Puebla	0.0000					
Oaxaca	0.0216	0.0000				
Querétaro	0.0250	0.0243	0.0000			
Guanajuato	0.0257	0.0257	0.0175	0.0000		
San Luis Potosí	0.0223	0.0173	0.0177	0.0185	0.0000	
Hidalgo	0.0211	0.0195	0.0193	0.0215	0.0153	0.0000

**Table 4:** Environmental variables of the natural habitats of wild populations of *Brickellia veronicifolia.*

Population	Precipitation (mm)			Temperature (°C)			Soil Physicochemical Properties				
	Aa	Max	Min	Aa	Max	Min	Moisture (%)	pH	P (mg/kg)	C (%)	N (mg/kg)
Puebla	56.0 ± 1.2	74.0 ± 0.9	44.3 ± 1.7	15.4 ± 0.7	27.9 ± 1.8	3.08 ± 1.1	8.769 ± 0.1 ^a^	7.623 ± 0.08 ^d^	0.057 ± 0.0 ^e^	0.251 ± 0.04 ^a^	12.129 ± 0.4 ^a^
Oaxaca	60.1 ± 1.6	129.0 ± 1.8	37.9 ± 1.3	18.8 ± 0.6	31.6 ± 1.3	6.8 ± 0.7	15.935 ± 0.2 ^ab^	8.203 ± 0.02 ^a^	0.044 ± 0.0 ^ab^	1.561 ± 0.04.^d^	50.933 ± 0.9 ^c^
Querétaro	41.4 ± 0.9	77.5 ± 0.7	5.8 ± 1.5	19.5 ± 1.3	30.8 ± 1.5	8.4 ± 1.5	34.775 ± 0.1 ^c^	8.060 ± 0.02 ^a^	0.040 ± 0.0 ^a^	1.005 ± 0.05 ^a^	9.353 ± 0.5 ^a^
Guanajuato	67.7 ± 2.3	90.3 ± 2.1	35.2 ± 1.0	19.6 ± 0.3	31.6 ± 0.6	7.9 ± 0.5	18.402 ± 0.1 ^a^	8.467 ± 0.02 ^e^	0.051 ± 0.0 ^f^	0.946 ± 0.04 ^a^	37.427 ± 0.8 ^bc^
San Luis Potosí	32.6 ± 1.4	55.5 ± 0.8	17.2 ± 1.8	17.5 ± 0.5	30.9 ± 0.7	3.2 ± 1.2	27.588 ± 0.3 ^ac^	4.493 ± 0.06 ^c^	0.150 ± 0.0 ^d^	0.394 ± 0.09 ^b^	7.018 ± 0.01 ^a^
Hidalgo	52.0 ± 1.7	67.5 ± 1.6	42.2 ± 1.4	15.1 ± 1.5	27.9 ± 1.0	1.5 ± 2.7	23.782 ± 0.5 ^ab^	6.113 ± 0.01 ^b^	0.135 ± 0.0 ^c^	3.225 ± 0.09 ^c^	28.066 ± 0.6 ^ab^

Aa: Annual average, Max: maximum, Min: minimum, ±*SD*: different letters in columns indicate statistically significant differences (Tukey test *p* ˂ 0.05).

**Table 5:** Location and reference vouchers of samples from *Brickellia veronicifolia.*

Population	Voucher Number	Altitude (masl)	Latitude	Longitude	Sample Size
Puebla	160,857	2472.4	19°24′35.1792″ N	−97°39′49.7946″ W	20
Oaxaca	160,842	1575.6	16°21′33.5334″ N	−96°36′11.556″ W	20
Querétaro	162,540	1829.8	20°9′3.1032″ N	−100°3′0.1332″ W	20
Guanajuato	162,537	1900.8	20°56′20.3928″ N	−101°19′56.8122″ W	20
San Luis Potosí	162,534	1973.2	22°9′6.7206″ N	−101°3′18.6372″ W	20
Hidalgo	162,541	2129.9	20°2′56.0328″ N	−99°25′16.7694″ W	20

## Data Availability

Not applicable.

## Supplementary Materials

The following are available online at https://www.mdpi.com/2223-7747/10/2/325/s1, Figure S1: *Brickellia veronicifolia* grown in xerophytic shrublands located in different provinces from Mexico, Figure S2: (A) Inflorescences of *B. veronicifolia* under wild conditions, (B) Winged achenes of *B. veronicifolia*, Figure S3: Monthly precipitation (mm; 2000–2017) for different populations of *B. veronicifolia* grown in Mexico, Figure S4: Monthly temperature variation (°C) from 2000–2017 by *B. veronicifolia* studied population, Table S1: Volatile constituents of the essential oil from *Brickellia veronicifolia* by population, Table S2: iPBS primers used in the detection of polymorphism among six populations of *Brickellia veronicifolia*.
