# Supplementary material for: Influence of Environmental Factors on the Genetic and Chemical Diversity of Brickellia veronicifolia Populations Growing in Fragmented Shrublands from Mexico
Source: Plants (Basel). 2021 Feb 8;10(2):325. doi: 10.3390/plants10020325 (PMC7915050; doi:10.3390/plants10020325)
Supplement: Supplementary file 1 [file plants-10-00325-s001.pdf]

SUPPLEMENTARY MATERIAL

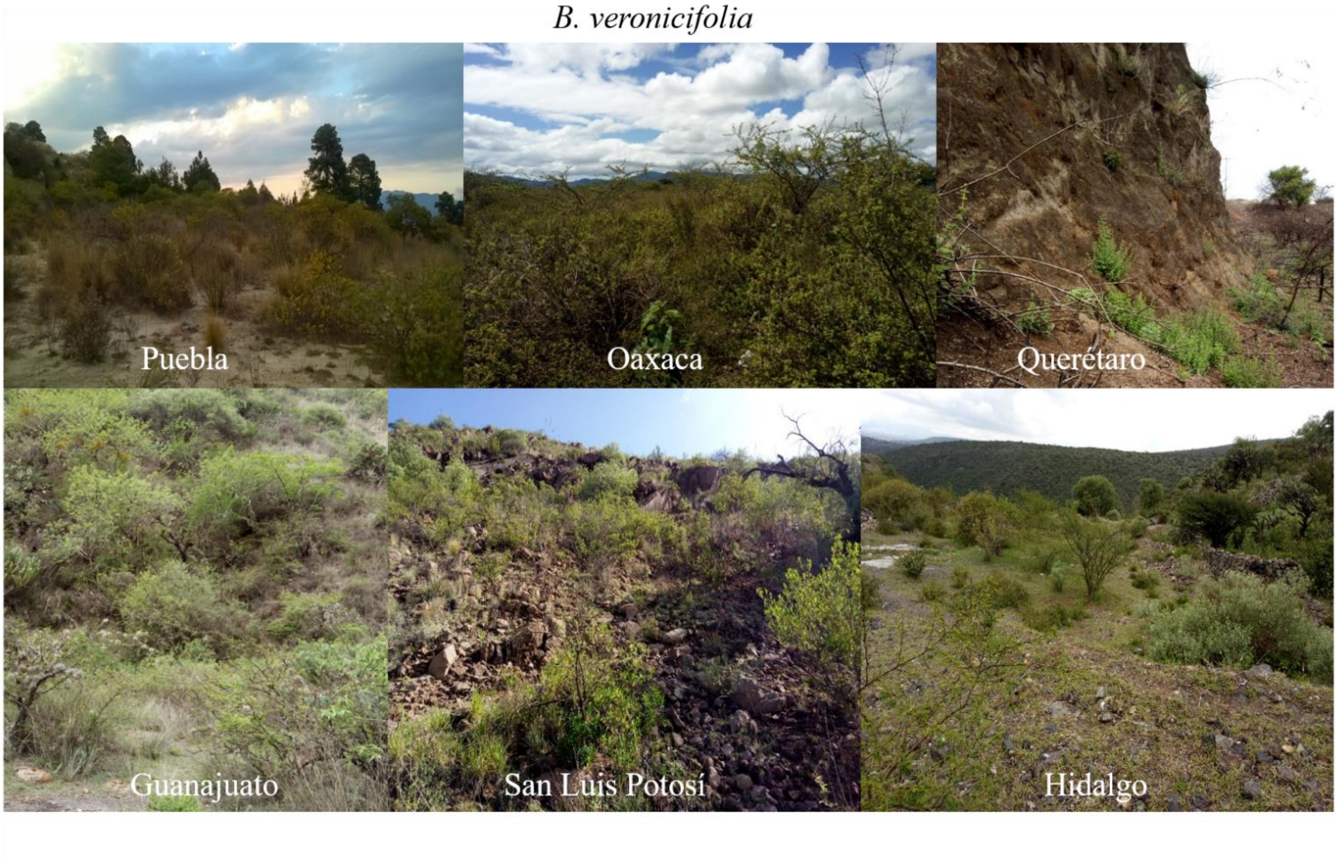

Figure S1: *Brickellia veronicifolia* grown in xerophytic shrublands located in different provinces from Mexico.

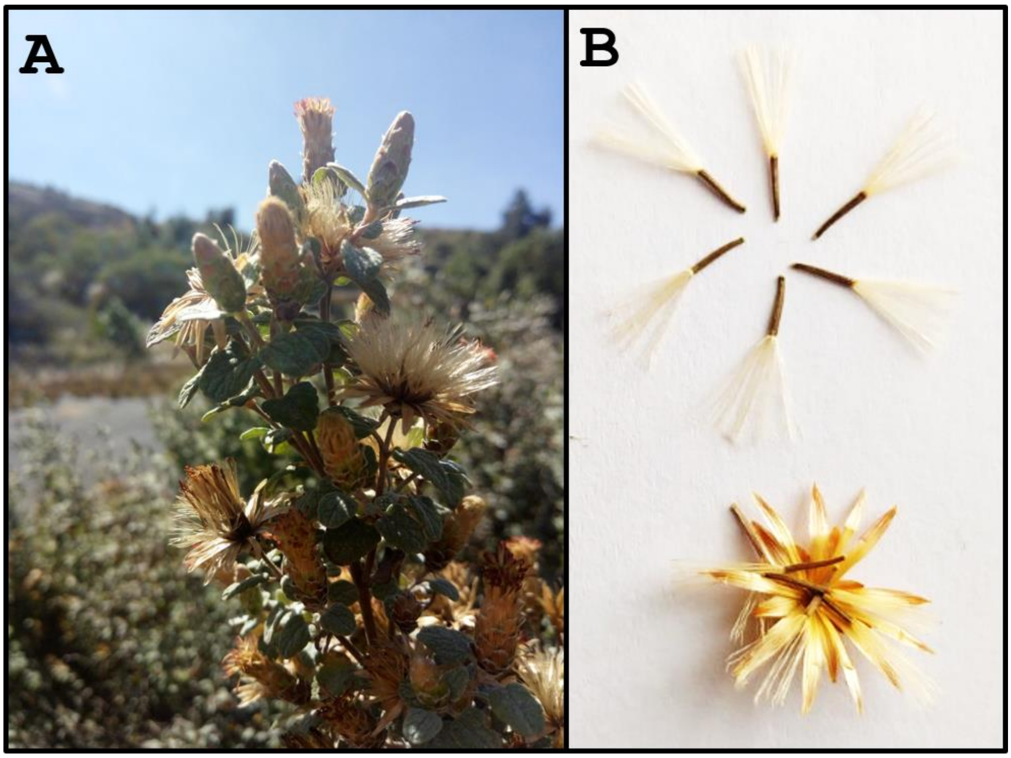

Figure S2: A) Inflorescences of *B. veronicifolia* under wild conditions, B) Winged achenes of *B. veronicifolia.*

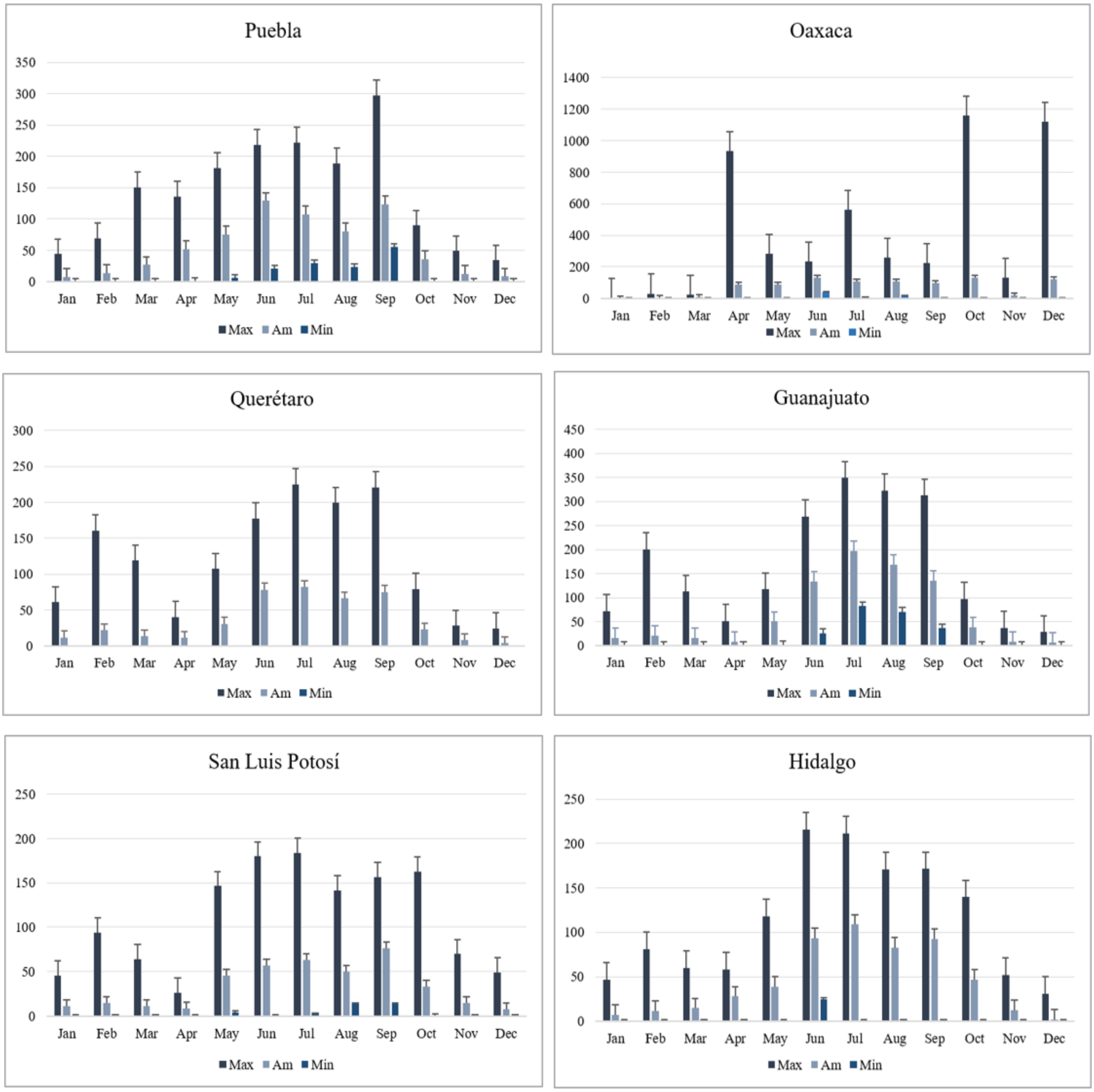

Figure S3: Monthly precipitation (mm; 2000-2017) for different populations of *B. veronicifolia* grown in Mexico (CONAGUA, 2018).

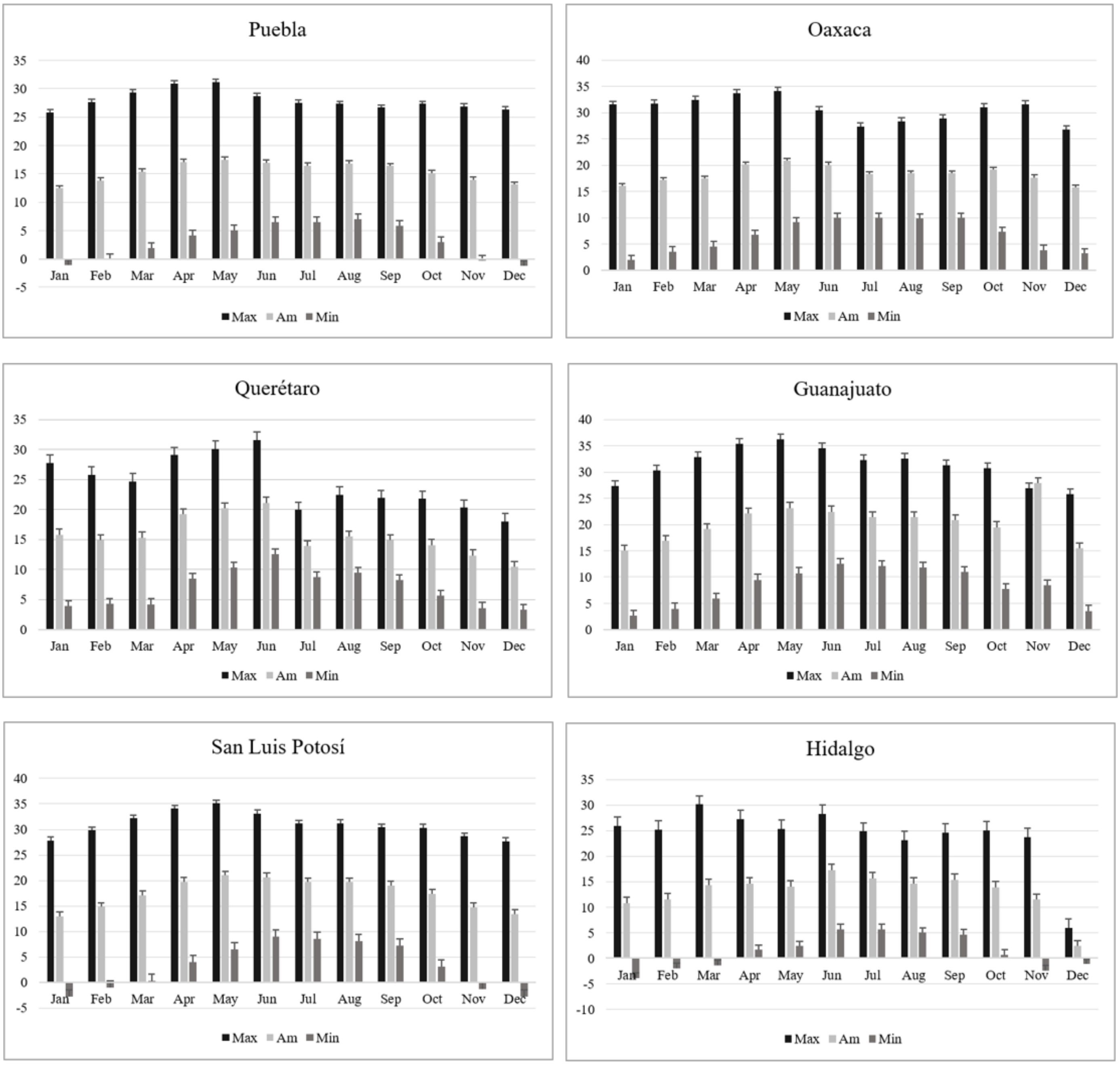

Figure S4: Monthly temperature variation (°C) from 2000-2017 by *B. veronicifolia* studied population (CONAGUA, 2018).

Table S1: Volatile constituents of the essential oil from *Brickellia veronicifolia* by population.

			PUEBLA			OAXACA			QUERETARO			GUANAJUATO			SAN LUIS POTOSÍ			HIDALGO		
# Peak	**RT mean**	**Compound**	**RRI-1**	**RRI-2**	**(%) RA**	**RRI-1**	**RRI-2**	**(%) RA**	**RRI-1**	**RRI-2**	**(%) RA**	**RRI-1**	**RRI-2**	**(%) RA**	**RRI-1**	**RRI-2**	**(%) RA**	**RRI-1**	**RRI-2**	**(%) RA**
**1**	8.50	D-Limonene	-	-	-	1024.2	1031.0	0.25±0.2	-	-	-	-	-	-	-	-	-	-	-	-
**2**	11.88	β-Linalool	1099.6	1098.0	0.08 ±0.4	1099.7	1098.0	0.09±0.2	1099.6	1098.0	0.07±0.0	1099.8	1098.0	0.06±0.0	1099.6	1098.0	0.11±0.0	1099.5	1098.0	0.11±0.0
**3**	12.43	Isopulegol	-	-	-	1117.6	1145.0	0.03±0.0	-	-	-	-	-	-	1118.1	1145.0	0.02±0.0	-	-	-
**4**	12.82	cis-Carvotanacetol	1166.8	1199.0	0.01±0.0	-	-	-	1117.8	1199.0	0.01±0.0	1117.5	1199.0	0.02±0.0	1117.8	1199.0	0.01±0.0	-	-	-
**5**	13.47	cis-Verbenol	-	-	-	-	-	-	1149.8	1142.0	0.07±0.0	-	-	-	1149.8	1142.0	0.05±0.0	1149.8	1142.0	0.02±0.0
**6**	13.98	cis-Carveol	1167.2	1229.0	0.01±0.0	-	-	-	1246.2	1229.0	0.02±0.0	1139.3	1229.0	0.02±0.0	1127.1	1229.0	0.01±0.0	-	-	-
**7**	14.18	(±)-Lavandulol	-	-	-	-	-	-	-	-	-	-	-	-	1172.2	1166.0	0.08±0.0	-	-	-
**8**	14.19	cis-Geraniol	-	-	-	-	-	-	1172.7	1240.0	0.02±0.0	1172.7	1240.0	0.01±0.0	1172.7	1240.0	0.03±0.0	1172.7	1240.0	0.02±0.0
**9**	14.20	cis-2-p-Menthen-1-ol	-	-	-	-	-	-	1173.0	1166.0	0.02±0.0	-	-	-	-	-	-	-	-	-
**10**	14.20	5-Caranol, trans,trans-(+)-	-	-	-	-	-	-	1173.0	1286.0	0.01±0.0	-	-	-	-	-	-	-	-	-
**11**	14.41	Terpinen-4-ol	1179.7	1177.0	0.01±0.0	-	-	-	1179.7	1177.0	0.05±0.0	1179.8	1177.0	0.02±0.0	1179.6	1177.0	0.14±0.0	1179.7	1177.0	0.06±0.0
**12**	14.60	p-Menth-8(10)-ene-2,9-diol	1185.9	1179.0	0.02±0.1	1176.5	1179.0	0.04±0.0	1186.0	1179.0	0.04±0.0	1186.0	1179.0	0.04±0.0	-	-	-	1185.9	1179.0	0.01±0.0
**13**	14.62	p-Cymen-8-ol	-	-	-	-	-	-	1186.2	1183.0	0.09±0.0	-	-	-	1186.3	1183.0	0.14±0.0	-	-	-
**14**	14.76	α-Terpineol	1190.9	1198.0	0.02±0.0	-	-	-	1191.1	1198.0	0.03±0.0	1191.0	1198.0	0.01±0.0	1191.0	1198.0	0.06±0.0	1191.0	1198.0	0.04±0.0
**15**	14.90	Myrtenol	-	-	-	-	-	-	1195.2	1194.0	0.06±0.0	-	-	-	1194.6	1194.0	0.14±0.0	1195.3	1194.0	0.05±0.0
**16**	15.09	endo-Borneol	-	-	-	-	-	-	1201.6	1165.0	0.03±0.0	-	-	-	-	-	-	-	-	-
**17**	15.22	Verbenone	-	-	-	-	-	-	1208.0	1204.0	0.05±0.0	1208.1	1204.0	0.05±0.0	1208.1	1204.0	0.07±0.0	1208.0	1204.0	0.04±0.0

**18**	15.22	Ascaridole epoxide	-	-	-	-	-	-	-	-	-	1208.1	1296.0	0.01±0.0	-	-	-	-	-	-
**19**	15.85	cis-3-Hexenyl isovalerate	1236.2	1241.0	0.02±0.1	-	-	-	1239.3	1241.0	0.04±0.0	1239.5	1241.0	0.02±0.0	1239.3	1241.0	0.04±0.0	1237.7	1241.0	0.02±0.0
**20**	16.01	D-Carvone	-	-	-	-	-	-	-	-	-	-	-	-	1246.0	1243.0	0.01±0.0	-	-	-
**21**	16.10	cis-3-Hexenyl valerate	-	-	-	-	-	-	-	-	-	-	-	-	-	-	-	1250.4	1230.0	0.06±0.0
**22**	16.15	p-Menthane-1,2,3-triol	-	-	-	-	-	-	1252.7	1485.0	0.02±0.0	1252.9	1485.0	0.01±0.0	-	-	-	-	-	-
**23**	16.69	β-citral	1273.0	1240.0	0.01±0.0	-	-	-	-	-	-	-	-	-	1273.0	1240.0	0.11±0.0	1279.5	1240.0	0.01±0.0
**24**	16.80	Carotol	-	-	-	-	-	-	1292.5	1549.0	0.02±0.0	-	-	-	-	-	-	-	-	-
**25**	17.57	Myrtenyl acetate	-	-	-	-	-	-	1327.0	1235.0	0.08±0.0	-	-	-	1327.3	1235.0	0.37±0.4	-	-	-
**26**	17.64	Isolongifolene	1335.9	1387.0	0.03±0.0	-	-	-	1327.3	1387.0	0.07±0.0	-	-	-	-	-	-	-	-	-
**27**	17.70	(+)-Sativen	-	-	-	1337.9	1339.0	0.02±0.0	1336.4	1339.0	0.02±0.0	1336.4	1339.0	0.02±0.0	1328.9	1339.0	0.03±0.0	1337.4	1339.0	0.02±0.0
**28**	17.81	γ-Elemene	1340.5	1430.0	1.10±1.7	1343.4	1430.0	0.05±0.0	1340.2	1430.0	0.06±0.0	1340.4	1430.0	0.09±0.0	-	-	-	1344.0	1430.0	2.84±1.6
**29**	18.00	α-Cubebene	-	-	-	1353.5	1351.0	0.92±1.1	1352.9	1351.0	0.37±0.2	1352.9	1351.0	0.09±0.2	-	-	-	1353.1	1351.0	0.47±0.2
**30**	18.06	α-Longipinene	1358.8	1351.0	0.38±0.3	-	-	-	-	-	-	-	-	-	1355.9	1351.0	0.94±1.1	-	-	-
**31**	18.16	β-Neoclovene	1363.1	1454.0	0.17±0.0	1363.0	1454.0	0.01±0.0	-	-	-	-	-	-	1362.6	1454.0	0.38±0.2	-	-	-
**32**	18.16	Isocaryophyllene	-	-	-	1363.2	1407.0	0.01±0.0	-	-	-	-	-	-	-	-	-	-	-	-
**33**	18.28	(+)- Longifolene	-	-	-	-	-	-	-	-	-	-	-	-	1370.5	1402.0	0.27±0.3	-	-	-
**34**	18.28	(+)-Cyclosativene	1370.3	1367.0	0.74±0.2	-	-	-	-	-	-	-	-	-	-	-	-	1370.3	1367.0	0.06±0.1
**35**	18.33	α-ylangene	1361.3	1382.0	1.02±1.4	1376.0	1382.0	0.16±0.1	1379.7	1382.0	1.82±0.0	1375.0	1382.0	0.06±0.0	1373.8	1382.0	0.11±0.0	1375.2	1382.0	0.01±0.0
**36**	18.26	Longicyclene	-	-	-	1363.1	1373.0	0.02±0.0	-	-	-	-	-	-	1374.9	1373.0	0.21±0.1	-	-	-
**37**	18.45	α-copaene	1381.7	1376.0	2.56±1.8	1380.4	1376.0	1.02±0.3	1384.1	1376.0	0.31±0.6	1379.6	1376.0	1.78±1.0	1379.4	1376.0	1.28±0.3	1379.6	1376.0	1.65±0.6
**38**	18.36	α-Gurjunene	1335.5	1409.0	0.14±0.0	1412.4	1409.0	0.18±0.0	-	-	-	-	-	-	-	-	-	1413.8	1409.0	0.33±0.0

**39**	18.48	β-Maaliene	1379.5	1380.0	0.21±0.1	-	-	-	1384.1	1380.0	0.31±0.1	1384.1	1380.0	0.36±1.1	-	-	-	1447.1	1380.0	0.03±0.1
**40**	18.50	β-Patchoulene	-	-	-	-	-	-	-	-	-	-	-	-	1384.1	1378.0	0.30±0.0	-	-	-
**41**	18.50	β-Chamigrene	-	-	-	1383.6	1475.0	0.76±0.0	-	-	-	-	-	-	-	-	-	-	-	-
**42**	18.49	(-)-β-Bourbonene	1357.1	1384.0	0.37±0.2	1389.7	1384.0	0.33±0.5	1388.2	1384.0	1.05±0.5	1389.2	1384.0	1.09±0.9	1388.4	1384.0	0.53±0.3	1388.7	1384.0	0.54±0.0
**43**	18.62	Berkheyaradulene	-	-	-	-	-	-	1391.0	-	1.24±0.3	1391.4	-	2.31±2.7	1390.8	-	0.80±0.2	1390.9	-	0.13±0.7
**44**	18.76	α-Muurolene	1372.1	1480.0	0.64±1.4	1370.7	1480.0	0.90±0.0	1403.6	1480.0	1.50±0.6	1370.3	1480.0	1.49±0.8	1470.3	1480.0	0.92±0.5	1397.2	1480.0	0.18±0.0
**45**	18.77	β-Elemene	-	-	-	1396.0	1391.0	0.78±0.0	1394.3	1391.0	1.26±0.4	1408.7	1391.0	11.17±8.2	-	-	-	-	-	-
**46**	18.81	Cedr-8-en-13-ol	-	-	-	-	-	-	1402.7	1668.0	0.03±0.0	-	-	-	1402.7	1668.0	0.19±3.6	-	-	-
**47**	19.15	β-Cubebene	1423.0	1390.0	6.30±4.7	1418.3	1390.0	0.56±0.0	-	-	-	1436.3	1390.0	3.12±1.5	-	-	-	1406.0	1390.0	1.04±3.3
**48**	19.15	Eremophilene	1394.1	1503.0	0.62±1.0	1447.1	1503.0	1.10±1.9	-	-	-	-	-	-	-	-	-	-	-	-
**49**	19.16	β-Caryophyllene	1413.8	1418.0	11.71±6.2	1424.8	1418.0	21.12±7.8	1422.7	1418.0	9.36±1.4	1422.6	1418.0	10.41±8.6	1422.3	1418.0	6.93±4.4	1423.5	1418.0	10.24±3.2
**50**	19.21	(±)-Cadinene	-	-	-	1424.4	1524.0	0.18±0.0	-	-	-	-	-	-	-	-	-	-	-	-
**51**	19.36	β-Guaiene	1364.1	1490.0	2.33±0.7	-	-	-	1397.2	1490.0	0.08±0.0	1369.0	1490.0	0.44±0.8	1411.0	1490.0	0.09±0.1	1537.5	1490.0	0.02±0.0
**52**	19.37	β-copaene	1447.1	1447.0	0.09±0.0	1431.4	1447.0	1.26±3.5	1430.1	1447.0	1.20±0.7	1430.3	1447.0	2.62±8.8	1421.0	1447.0	0.58±0.6	1439.9	1447.0	0.03±0.0
**53**	19.43	γ-Gurjunene	-	-	-	-	-	-	-	-	-	-	-	-	-	-	-	1436.4	1473.0	0.01±0.0
**54**	19.43	α-Bergamotene	-	-	-	-	-	-	-	-	-	-	-	-	1435.2	1434.0	0.23±0.2	-	-	-
**55**	19.41	Di-epi-α-cedrene	-	-	-	1435.5	1375.0	0.05±0.7	-	-	-	-	-	-	-	-	-	-	-	-
**56**	19.42	7-epi-cis-sesquisabinene hydrate	-	-	-	-	-	-	-	-	-	1435.9	1660.0	0.04±0.0	-	-	-	-	-	-
**57**	19.44	3,7(11)-Selinadiene	-	-	-	-	-	-	-	-	-	-	-	-	-	-	-	1436.8	1532.0	0.37±0.0
**58**	19.45	Alloaromadendrene	1462.9	1461.0	0.49±0.7	1461.7	1461.0	0.25±0.2	1462.6	1461.0	0.23±0.0	1462.6	1461.0	0.0±0.0	-	-	-	1463.2	1461.0	0.26±0.1
**59**	19.36	β-Gurjunene	-	-	-	1432.7	1432.0	0.19±0.0	-	-	-	-	-	-	-	-	-	-	-	-

**60**	19.51	Aromandendrene	1440.5	1440.0	0.45±0.0	1441.4	1440.0	0.70±0.5	1440.2	1440.0	0.34±0.1	1444.4	1440.0	0.77±0.8	1440.2	1440.0	0.26±0.2	1440.6	1440.0	0.37±0.0
**61**	19.78	Humulene	1455.5	1440.0	1.55±0.7	1459.6	1440.0	1.82±0.7	1455.2	1440.0	1.28±0.4	1455.1	1440.0	0.89±0.9	1455.2	1440.0	0.90±0.5	1455.7	1440.0	1.64±0.8
**62**	20.12	γ-Muurolene	1480.4	1477.0	1.20±1.7	1454.1	1477.0	3.59±1.4	1478.7	1477.0	1.77±0.5	1478.6	1477.0	1.01±0.9	1478.6	1477.0	1.33±0.5	1479.9	1477.0	0.48±1.6
**63**	20.44	(+)-Ledene	1487.4	1493.0	0.38±0.2	1441.7	1493.0	1.19±2.0	1485.3	1493.0	0.16±0.1	1435.9	1493.0	0.01±0.0	1468.8	1493.0	0.36±0.0	1487.3	1493.0	0.37±5.6
**64**	20.50	α-Curcumene	-	-	-	1484.7	1483.0	2.14±0.1	-	-	-	-	-	-	1505.4	1483.0	1.74±0.8	-	-	-
**65**	20.64	γ-Elemene	-	-	-	-	-	-	-	-	-	-	-	-	-	-	-	1502.2	1430.0	0.92±3.7
**66**	20.63	Cubedol	1501.0	1580.0	5.59±4.4	1500.6	1580.0	4.54±0.8	1501.1	1580.0	7.15±6.1	1500.3	1580.0	6.51±6.6	1500.7	1580.0	7.45±4.5	1505.6	1580.0	9.07±3.7
**67**	20.84	γ-Cadinene	1516.9	1513.0	1.85±1.7	1518.4	1513.0	4.55±2.6	-	-	-	-	-	-	1516.4	1513.0	1.94±0.5	1484.3	1513.0	18.32±2.2
**68**	20.86	α-Himachalene	-	-	-	1511.6	1447.0	0.25±0.2	-	-	-	-	-	-	1509.7	1447.0	0.30±0.1	-	-	-
**69**	21.19	trans-calamenene	-	-	-	-	-	-	-	-	-	1523.6	1528.0	1.27±0.7	1524.1	1528.0	1.32±0.7	-	-	-
**70**	21.21	(+)-δ-Cadinene	1524.6	1524.0	5.40±2.4	1525.4	1524.0	2.44±1.5	1525.9	1524.0	1.88±0.4	1526.1	1524.0	1.93±1.1	-	-	-	1524.4	1524.0	3.03±3.7
**71**	21.37	α-acorenol	-	-	-	-	-	-	1509.2	1629.0	0.13±0.0	1340.6	1629.0	0.02±0.0	1645.4	1629.0	2.11±1.4	1554.5	1629.0	0.79±0.3
**72**	21.55	γ-Himachalene	-	-	-	-	-	-	-	-	-	-	-	-	1537.8	1475.0	0.37±0.2	-	-	-
**73**	21.54	α-Cadinene	1537.1	1538.0	0.56±0.2	1537.1	1538.0	0.14±0.0	-	-	-	-	-	-	-	-	-	1537.8	1538.0	1.18±0.1
**74**	21.74	4-epi-cubedol	1550.4	1494.0	1.45±2.5	1525.3	1494.0	0.18±0.1	1559.6	1494.0	0.20±0.1	1534.8	1494.0	0.63±0.2	1582.6	1494.0	0.19±0.1	1531.2	1494.0	0.02±0.0
**75**	21.81	Murolan-3,9(11)-diene-10-peroxy	-	-	-	-	-	-	-	-	-	-	-	-	1548.1	1730.0	0.46±1.0	-	-	-
**76**	21.88	Longiverbenone	-	-	-	-	-	-	1541.2	2148.0	0.76±0.7	1560.5	2148.0	0.08±0.1	-	-	-	-	-	-
**77**	22.04	6-epi-shyobunol	-	-	-	-	-	-	-	-	-	-	-	-	1319.1	1517.0	0.01±0.0	1691.1	1517.0	1.77±0.2
**78**	22.16	Nerolidyl acetate	-	-	-	-	-	-	1562.1	1675.0	1.27±0.4	1561.5	1675.0	0.74±0.2	-	-	-	1561.8	1675.0	0.07±0.3
**79**	22.27	Nerolidol	-	-	-	-	-	-	1565.1	1560.0	0.14±0.2	-	-	-	1564.7	1560.0	1.07±0.7	1569.0	1560.0	0.18±0.3
**80**	22.34	Aristolene epoxide	1568.7	1747.0	0.14±0.4	-	-	-	-	-	-	-	-	-	-	-	-	-	-	-
**81**	22.35	Epiglobulol	-	-	-	-	-	-	-	-	-	-	-	-	1569.4	1588.0	0.88±0.7	-	-	-

**82**	22.51	Globulol	1576.0	1576.0	0.33±0.4	1583.1	1576.0	0.70±0.0	1576.8	1576.0	1.75±0.6	1568.4	1576.0	0.79±0.4	1574.8	1576.0	0.76±0.5	1571.7	1576.0	8.08±4.4
**83**	22.77	(-)-Spathulenol	1584.6	1576.0	13.74±5.8	1586.7	1576.0	18.34±6.7	1585.2	1576.0	14.93±1.4	1583.6	1576.0	11.47±4.5	1587.0	1576.0	11.17±5.8	1586.5	1576.0	7.44±3.4
**84**	22.91	Caryophyllene oxide	1590.2	1581.0	9.82±2.2	1592.2	1581.0	15.95±2.8	1590.1	1581.0	20.14±3.9	1589.4	1581.0	19.31±5.0	1592.0	1581.0	15.73±11.9	1592.9	1581.0	2.92±5.5
**85**	23.11	Ledol	1597.8	1565.0	1.28±0.3	1598.9	1565.0	0.90±0.6	1598.0	1565.0	1.80±0.9	1597.2	1565.0	0.90±0.5	1599.2	1565.0	1.74±1.0	1600.5	1565.0	2.02±0.3
**86**	23.22	Guaiol	-	-	-	-	-	-	-	-	-	1602.2	1595.0	0.16±0.3	-	-	-	-	-	-
**87**	23.43	Viridiflorol	1607.5	1590.0	1.10±0.7	-	-	-	1607.3	1590.0	1.93±1.0	-	-	-	-	-	-	1608.3	1590.0	0.28±0.8
**88**	23.49	Humulene-1,6-dien-3-ol	-	-	-	-	-	-	-	-	-	-	-	-	-	-	-	1609.1	1619.0	0.61±1.2
**89**	23.67	Calarene epoxide	1612.9	1671.0	0.24±0.7	-	-	-	1613.3	1671.0	0.50±0.7	1615.3	1671.0	0.11±0.1	-	-	-	-	-	-
**90**	23.88	Drimenol	-	-	-	-	-	-	1619.2	1752.0	0.86±0.2	-	-	-	1619.4	1752.0	0.83±0.4	1619.8	1752.0	0.09±0.3
**91**	24.14	Diepicedrene-1-oxide	-	-	-	1614.0	1548.0	0.24±0.7	1611.8	1548.0	2.12±1.2	1611.2	1548.0	0.80±1.0	1612.0	1548.0	2.56±1.5	1681.9	1548.0	1.00±0.1
**92**	24.05	9-Methoxycalamenene	1542.5	1484.0	0.61±0.3	-	-	-	1664.8	1484.0	0.53±0.3	1664.9	1484.0	0.42±0.2	1666.2	1484.0	0.47±0.2	1542.7	1484.0	0.85±0.0
**93**	24.31	β-Ionol	-	-	-	-	-	-	1630.8	1406.0	0.30±0.1	1630.3	1406.0	0.42±0.1	-	-	-	-	-	-
**94**	24.61	Alloaromadendrene oxide	1624.6	1625.0	0.62±0.6	1669.9	1625.0	0.14±0.0	1625.1	1625.2	0.95±0.8	-	-	-	1606.3	1625.0	0.27±0.4	-	-	-
**95**	24.67	tau.-Muurolol	1640.5	1640.0	8.88±5.6	1638.1	1640.0	2.57±5.6	1640.6	1640.0	6.01±3.0	1640.4	1640.0	4.11±3.6	1639.6	1640.0	5.99±4.6	1640.8	1640.0	1.28±3.8
**96**	24.73	tau-cadinol	1641.1	1642.0	1.80±5.5	1642.8	1642.0	4.82±1.6	-	-	-	-	-	-	1640.8	1642.0	11.67±3.4	-	-	-
**97**	24.77	(-)-Isolongifolol	1636.9	1723.0	0.52±0.5	1630.5	1723.0	0.81±0.8	1663.5	1723.0	0.52±0.2	1640.6	1723.0	1.32±0.5	1644.5	1723.0	1.00±2.5	1662.4	1723.0	0.77±0.2
**98**	24.80	(-)-δ-Cadinol	-	-	-	-	-	-	-	-	-	-	-	-	1643.2	1636.0	1.43±1.0	-	-	-
**99**	24.83	δ-Cedrol	1644.2	1621.0	1.37±0.8	1643.4	1621.0	0.75±0.8	1643.8	1621.0	2.88±2.6	1644.9	1621.0	2.40±2.3	1644.3	1621.0	1.43±0.7	1644.7	1621.0	2.20±1.0
**100**	25.01	Z-9-Pentadecenol	-	-	-	-	-	-	1648.3	1742.0	1.25±0.4	1648.4	1742.0	0.61±0.5	1648.5	1742.0	0.66±0.3	1650.0	1742.0	2.24±0.2
**101**	25.15	α-Cadinol	1654.6	1653.0	9.91±1.7	1653.2	1653.0	1.694±2.3	1652.5	1653.0	7.18±3.3	1652.3	1653.0	5.96±3.2	1650.8	1653.0	5.80±1.9	1651.7	1653.0	11.68±3.5
**102**	25.75	Longifolenaldehyde	1603.9	1601.0	1.26±0.5	1604.3	1601.0	0.10±0.0	1602.4	1601.0	0.70±0.3	1600.5	1601.0	0.36±0.2	1717.6	1601.0	0.16±0.1	1662.2	1601.0	0.44±0.2

**103**	25.84	Patchoulol	-	-	-	-	-	-	-	-	-	-	-	-	1670.5	1664.0	1.02±0.5	-	-	-
**104**	26.25	α-Bisabolol	-	-	-	-	-	-	-	-	-	1681.0	1683.0	0.29±0.2	1681.5	1883.0	0.40±0.4	1681.3	1683.0	0.11±0.3
**105**	26.97	Aromadendrene oxide	1747.4	1702.0	0.20±0.0	1728.9	1702.0	0.19±0.4	-	-	-	1748.4	1702.0	0.68±0.4	1767.8	1702.0	0.31±0.1	1662.2	1702.0	1.09±0.4
**106**	29.25	Corymbolone	1740.2	1892.0	0.35±0.2	1721.7	1892.0	0.83±0.1	1737.8	1892.0	0.37±0.3	-	-	-	1738.1	1892.0	0.12±0.2	1800.9	1892.0	0.02±0.0
**107**	30.23	cis-Z-α-Bisabolene epoxide	-	-	-	1778.6	1743.0	0.14±0.3	1777.2	1743.0	0.59±0.1	1777.4	1743.0	0.54±0.2	1775.9	1743.0	0.55±0.1	-	-	-
**108**	33.29	Geranyl-p-cymene	1959.1	2006.0	0.36±0.2	-	-	-	-	-	-	-	-	-	1959.1	2006.0	0.07±0.0	1959.1	2006.0	0.03±0.1

Compounds identified by the estimation of RRI-1 and RRI-2, relative retention index for DB−5 and HP−5 columns, respectively. (%) RA: relative abundance. A dash (−) indicates the absence of the component. Shadow areas show the 6 more abundant compounds and enclosed are the specific compounds of each population.

Table S2: iPBS primers used in the detection of polymorphism among six populations of *Brickellia veronicifolia.*

iPBS Marker	Sequence (*5*'–*3*')	Ta (°C)
**1846**	CTGGCATTTCCATTGTCGTCGATGC	55.5
**1880**	GAACTCCCTGGTGGCATCGTGAGC	60.5
**2074**	GCTCTGATACCA	50.0
**2075**	CTCATGATGCCA	51.2
**2076**	GCTCCGATGCCA	49.0
**2077**	CTCACGATGCCA	50.5
**2078**	GCGGAGTCGCCA	55.0
**2080**	CAGACGGCGCCA	55.0
**2081**	GCAACGGCGCCA	58.0
**2232**	AGAGAGGCTCGGATACCA	53.0
**2249**	AACCGACCTCTGATACCA	51.0
**2378**	GGTCCTCATCCA	53.0

Ta: alignment temperature
